# Adaptations of membrane trafficking in cancer and tumorigenesis

**DOI:** 10.1242/jcs.260943

**Published:** 2024-05-21

**Authors:** Emma Evergren, Ian G. Mills, Grace Kennedy

**Affiliations:** ^1^ Patrick G. Johnston Centre for Cancer Research, Queen's University Belfast, 97 Lisburn Road, Belfast BT9 7BL, UK; ^2^ Nuffield Department of Surgical Sciences, University of Oxford, Oxford OX3 9DU, UK

**Keywords:** Endocytosis, Cancer, Coatomer, Imaging, Vesicle

## Abstract

Membrane trafficking, a fundamental cellular process encompassing the transport of molecules to specific organelles, endocytosis at the plasma membrane and protein secretion, is crucial for cellular homeostasis and signalling. Cancer cells adapt membrane trafficking to enhance their survival and metabolism, and understanding these adaptations is vital for improving patient responses to therapy and identifying therapeutic targets. In this Review, we provide a concise overview of major membrane trafficking pathways and detail adaptations in these pathways, including COPII-dependent endoplasmic reticulum (ER)-to-Golgi vesicle trafficking, COPI-dependent retrograde Golgi-to-ER trafficking and endocytosis, that have been found in cancer. We explore how these adaptations confer growth advantages or resistance to cell death and conclude by discussing the potential for utilising this knowledge in developing new treatment strategies and overcoming drug resistance for cancer patients.

## Teaser

**Summary:** Cancer cell proliferation and tumorigenesis rely on adaptations in membrane trafficking, which play crucial roles in nutrient availability, metabolic regulation and resistance to therapy as reviewed here.

## Introduction

Cancer cells undergo significant adaptations in basic cell functions to meet their energetic needs and upregulate anti-apoptotic and cell proliferative mechanisms. An important enabler of these adaptations is membrane trafficking. Membrane trafficking mediates many processes including subcellular distribution of proteins, termination of pro-proliferative signalling from the plasma membrane, autophagy, migration, and secretion of enzymes that cancer cells use to promote tumorigenesis, facilitate invasion and acquire drug resistance. Although adaptation of membrane trafficking itself is not a classical cancer hallmark, it directly impacts many of the hallmarks of cancer as defined by Hanahan and Weinberg, and thereby promotes cancer progression (Hanahan, 2022; Hanahan and Weinberg, 2000).

**Fig. 1.**
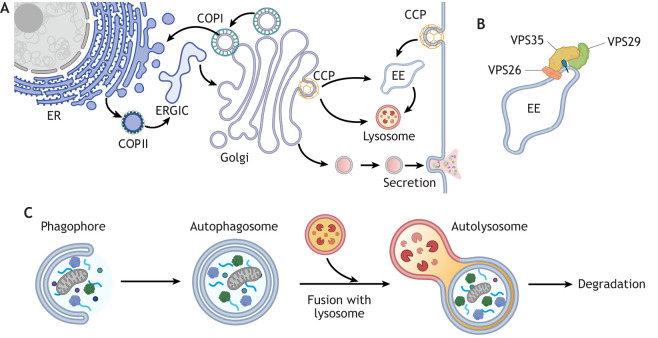
**Overview of intracellular membrane trafficking pathways.** (A) Vesicle trafficking pathways transport cargo to and from the plasma membrane, ER, Golgi, endosomes and lysosomes. Trafficking of proteins and lipids between organelles is fundamental for intracellular signalling, secretion and maintenance of organelle identity and compartmentalisation of cellular functions (Escribá et al., 2015; Haucke and Paolo, 2007; Kozlov and Taraska, 2023; Yang et al., 2018a). Here, three types of coated vesicles and their trafficking pathways are shown. COPII-coated vesicles bud from ER exit sites and fuse with the ER-Golgi intermediate compartment (ERGIC) (Raote et al., 2022); COPI-coated vesicles mediate retrograde trafficking within the Golgi and to the ER; and clathrin-coated pits (CCP) mediate export of vesicles from the Golgi to early endosomes (EE) and lysosomes. In addition, CCPs generate clathrin-coated vesicles at the plasma membrane, which traffic through the endolysosomal system and recycling pathways. (B) Trafficking from the early endosome is regulated by the retromer complex. The core retromer complex is composed of a heterotrimer of a VPS26 protein, VPS29 and VPS35 that recognises cargo receptors on the endosomal membrane (Seaman et al., 1997, 1998). (C) Autophagy (macroautophagy) is a membrane trafficking pathway that acts as an important catabolic mechanism for generating energy during nutrient starvation and in cells with higher metabolic rates, such as cancer cells. A membrane cup called the phagophore engulfs the cytosolic contents or obsolete organelles to form an autophagosome, which subsequently fuses with a lysosome, at which stage membranes and proteins are degraded by lysosomal hydrolases. Autophagy intersects with multiple membrane trafficking pathways, including the COPII, COPI and endocytic pathways.

Membrane trafficking pathways comprise fundamental cellular processes that contribute to cell–cell communication, internalisation of cargo, secretion of proteins, inter-organelle communication and signalling. Vesicular trafficking between organelles is mediated by coat protein complexes, cargoes and accessory proteins that together drive formation of vesicles (Kozlov and Taraska, 2023; McMahon and Mills, 2004; Mehrani and Stagg, 2022). Endocytosis and endosomal trafficking from the plasma membrane enable uptake of nutrients from the extracellular environment and regulate expression of cell surface receptors involved in growth factor signalling (Fig. 1A). Endocytosed cargo is either trafficked to lysosomes for degradation or to the endosome for recycling. The recycling route from endosomes to the plasma membrane and the trans-Golgi network, controlled by the retromer complex, orchestrates cargo selection and formation of tubulovesicular carriers (Seaman, 2019) (Fig. 1B). Endoplasmic reticulum (ER)-to-Golgi anterograde trafficking is dependent on the formation of COPII coatomer-coated vesicles at the ER, which sustains the capacity of the secretory pathway and ensures that newly synthesised proteins in the ER are subsequently appropriately folded and, in the case of membrane-bound proteins, glycosylated in the Golgi. Golgi-to-ER retrograde trafficking is mediated by COPI-coated vesicles, ensures proper localisation of ER-resident proteins that regulate the fidelity of protein synthesis in the ER (Gomez-Navarro and Miller, 2016) and also plays an important role in autophagic flux (Claerhout et al., 2012). Thus, membrane trafficking proteins also mediate macroautophagy (hereafter simply called autophagy). Autophagy is a conserved, lysosome-dependent pathway that degrades and recycles cellular components and is activated by nutrient deprivation and oxidative stress (Fig. 1C). Although there is a distinct set of proteins that facilitate the formation of autophagosome biogenesis, there is a significant crosstalk between autophagic proteins and other membrane trafficking components (Claerhout et al., 2012; Li et al., 2020; Viret and Faure, 2019).

Cells acquire tumorigenic potential through a process called transformation, during which significant adaptions due to signalling and transcriptional changes occur. These adaptations can result in alterations in the expression of vesicle coat and accessory proteins (Table 1) and the lipid composition of membranes (Box 1). Together, these changes promote oncogenic signalling, cell survival and proliferation (Lee et al., 2023). In this Review, we will discuss how alterations of membrane trafficking between core organelles contribute to cancer progression, therapy resistance and the efficacy of drug delivery. The role of membrane trafficking in cancer cell migration is beyond the scope of this Review and has been recently covered in other excellent review articles (Sigismund et al., 2021; Yayan et al., 2024). Here, we will focus on how cancer cells utilise membrane trafficking to control nutrient availability and metabolic activity, regulate receptor signalling and control secretion. Furthermore, we will discuss how these adaptations contribute to therapy resistance and how we potentially can harness membrane trafficking to treat and stratify cancer patients in the future.

**Box 1. Phosphoinositides are central regulators of membrane trafficking**

Alterations in lipid biosynthesis correlate with disease progression in a number of cancer types, and with tumour progression in prostate, breast and liver cancer (Butler et al., 2016, 2021; Dadhich and Kapoor, 2022; Khan et al., 2021; Tan et al., 2022; Ward et al., 2021; Zalba and ten Hagen, 2017; Zhang et al., 2020a). The lipid composition of cellular membranes is fundamental to cell compartmentalisation and organelle identity. In particular, phosphoinositides (PIs) are concentrated in a compartment-specific manner and constitute an important identifier for each organelle (Haucke and Paolo, 2007; Hein et al., 2023 preprint).

The lipid composition of different organelles facilitates recruitment of effector proteins that have membrane-binding domains with specificity for different species of PI (reviewed in Davies et al., 2023; Paolo and Camilli, 2006). Mutations and amplifications in PI kinases and phosphatases found in cancer therefore impact on membrane trafficking and diverse cellular functions. For example, phosphatidylinositol-5-phosphate 4-kinases (PI5P4Ks) are upregulated in breast and prostate cancer where they regulate cell survival and tumorigenesis (Emerling et al., 2013; Triscott et al., 2023).

Phosphatidylinositol 4-phosphate 5-kinases (PI4P5Ks), which catalyse the five-phosphorylation of PI(4)P to generate PI(4,5)P_2_, are another example of PI kinases overexpressed in breast and advanced prostate cancer (Sarwar et al., 2019; Semenas et al., 2014). PI(4,5)P_2_ is predominantly found on the plasma membrane where it aids in the initiation of clathrin-mediated endocytosis and activates talin proteins to induce formation of invadopodia (Kadlecova et al., 2017). Thus, alterations in lipid composition of different organelles and PI-associated enzymes influence major cellular functions and are likely to be highly relevant in cancer pathogenesis and for potential therapeutic interventions.

**Table 1.**
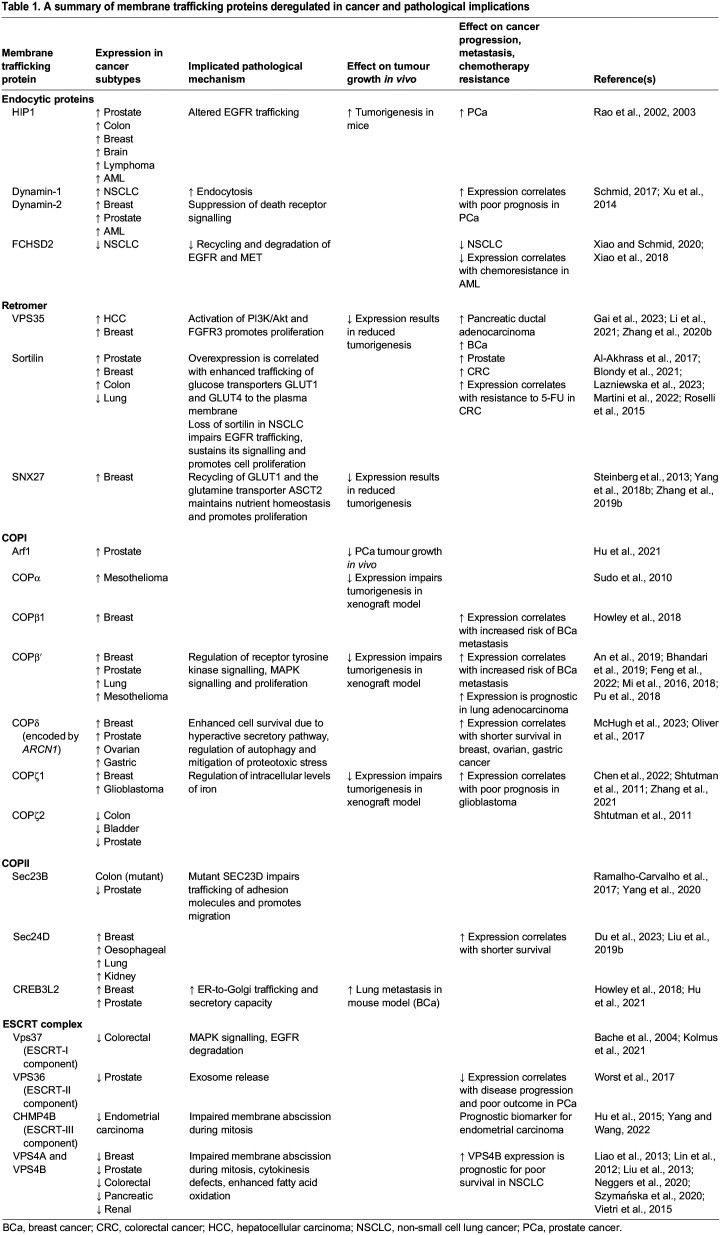
A summary of membrane trafficking proteins deregulated in cancer and pathological implications

## Adaptations in endocytosis support tumour growth and metabolism reprogramming

The abnormal growth of tumour cells relies on the activation of pathways promoting cell survival, proliferation and metabolism. Among these, regulatory mechanisms governing membrane trafficking of receptors and transporters to and from the cell surface are increasingly recognised as crucial for supporting uncontrolled tumour cell growth. In response to the heightened energy demands associated with uncontrolled growth, cancer cell metabolism undergoes reprogramming, most prominently observed through the Warburg effect, in which aerobic glycolysis is employed for the production of ATP from glucose, and through increased lipid metabolism via elevated lipogenesis and lipid uptake (Munir et al., 2019).

Cancer cells show flexibility and adaptability in their endocytic capacity in order to sustain mechanisms that support nutrient scavenging of lipoproteins, glucose and transferrin. Important mechanisms of nutrient uptake include macropinocytosis and clathrin-mediated endocytosis. Clathrin-mediated endocytosis is the best-characterised form of endocytosis and functions as both a constitutive and ligand-induced pathway for internalisation of cargo from the plasma membrane. Increased growth signalling in tumour cells sustains proliferation and accelerated growth, and this can involve upregulation of growth factors and receptors on the cell surface or acquisition of mutations that render these receptors constitutively active (Hanahan and Weinberg, 2000). Growth factor receptors belong to the receptor tyrosine kinase (RTK) family. Examples of receptors that are overexpressed in multiple cancer types include epidermal growth factor receptor (EGFR), fibroblast growth factor receptor (FGFR) and mesenchymal-epithelial transition factor (MET). Dysregulated RTK activation and signalling triggers RTK-induced oncogenesis through a wide range of signalling pathways including the phosphoinositide 3-kinase (PI3K)-AKT, JAK2-STAT and mitogen-activated protein kinase (MAPK) pathways (Du and Lovly, 2018). Recent research underscores the significance of spatial regulation of RTKs in proliferative signalling and resistance to RTK-targeted therapies (Casaletto and McClatchey, 2012). Consequently, the trafficking of receptors to and from the plasma membrane has emerged as a crucial factor in understanding the regulation of RTK signalling (Mellman and Yarden, 2013; Sigismund et al., 2021). For example, clathrin-mediated endocytosis is downregulated in multiple non-small cell lung cancer (NSCLC) cell lines compared to what is seen in normal lung epithelial cells (Elkin et al., 2015). The dynamics and life-time of clathrin-coated pits are regulated by the tumour suppressor phosphatase and tensin homolog (PTEN) in breast cancer cell lines (Rosselli-Murai et al., 2018) and the prevalence of flat clathrin lattices correlates with metastatic potential in colorectal cell lines (Cresens et al., 2023). Moreover, upregulated expression of some endocytic proteins, such as HIP1 and dynamin-1 and -2, has been found to be pro-proliferative or anti-apoptotic (Table 1). Endocytic control of growth factor signalling has been previously discussed in several excellent reviews (Barbieri et al., 2016; Caldieri et al., 2018; Mosesson et al., 2008; Sigismund et al., 2021). Here, we will focus on endocytic mechanisms involved in the uptake of nutrients that promote cancer cell survival and tumorigenesis.

### The retromer complex regulates glucose transporters in cancer cells

Metabolic reprogramming in cancer cells facilitates the increased energy production associated with tumour growth and includes alterations in both the metabolism and uptake of glucose and lipids. Endocytosis and recycling of cell surface receptors is vital for regulating internalisation of lipids and glucose, which are fundamental to sustaining energy production, membrane biogenesis and protein synthesis. Failure to accurately sort and recycle receptors has been linked to a number of human diseases including cancer (Carosi et al., 2023). Some retromer-associated proteins, such as sorting nexin-27 (SNX27) and sortilin, are overexpressed and correlate with cancer progression and metastasis in prostate, liver, colorectal and breast cancer (Table 1) (Berger et al., 2021; Blondy et al., 2021; Chan et al., 2023; Gao et al., 2022; Martini et al., 2022; Sharma et al., 2019; Ye et al., 2022).

SNX27 promotes cell proliferation, migration and tumorigenesis by positively regulating cellular energy homeostasis and the trafficking of proteases (Deb and Sun, 2022). SNX27–retromer complexes directly interact with the glucose transporter GLUT1 and facilitate its recycling to the plasma membrane, which promotes glucose uptake and cell survival (Steinberg et al., 2013). Under normal conditions, this interaction is inhibited by the tumour suppressor PTEN, resulting in fewer GLUT1 channels present at the cell surface and a reduced glucose uptake (Shinde and Maddika, 2017). Mutation or loss of PTEN is associated with tumorigenesis in a range of tissues (Salmena et al., 2008). For example, a somatic mutation of PTEN (T401I) associated with soft tissue sarcoma inhibits its interaction with SNX27, resulting in reduced retromer-mediated recycling and accumulation of GLUT1 at the plasma membrane (Shinde and Maddika, 2017). In prostate cancer cells *in vitro*, elevated glucose concentrations have been shown to lead to an increase in sortilin protein expression, whereas glucose deprivation has been shown to cause a reduction in its expression, demonstrating that the retromer undergoes an adaptive response to metabolic changes (Lazniewska et al., 2023). In prostate cancer, which is a hormone-dependent cancer, androgens increase glucose uptake (Vaz et al., 2016; White et al., 2018), and increase expression of both sortilin and GLUT1, which leads to enhanced trafficking of GLUT1 to the plasma membrane (Lazniewska et al., 2023). Further unravelling the mechanisms of retromer-mediated trafficking will be imperative for a comprehensive understanding of its role in the regulation of metabolic reprogramming, particularly in cancer cells.

### Iron homeostasis regulates cancer cell survival and growth

Iron and iron-binding proteins are essential to sustaining cell proliferation through regulation of mitochondrial metabolism, DNA synthesis and repair, and lysosomal activity (Galy et al., 2024; Weber et al., 2020). Iron ions are transported by transferrin and constitutively internalised by the transferrin receptor. Once internalised, iron is trafficked through the endo-lysosomal system, where it is processed, after which free iron ions are released (Shen et al., 2018). Cancer cells adapt iron homeostasis to support an increased need for biosynthesis and energy, and the number of transferrin receptors present on a cell strongly correlates with the rate of cell proliferation (Chitambar et al., 1983; Neckers and Trepel, 1986; Richardson, 2002). Upregulation of the transferrin receptor is associated with poor outcomes in a range of cancer types, including breast cancer (Khoo et al., 2020), leukaemia (Hagag et al., 2018) and advanced prostate cancer (O'Donnell et al., 2006). However, excess intracellular non-transferrin-bound iron can result in toxicity due to formation of oxygen radicals and damage to lipids and DNA, which creates a potential vulnerability in tumour cells (Galy et al., 2024). The iron-dependent peroxidation of lipids, particularly phospholipids, is hallmark of ferroptosis, a non-apoptotic form of cell death in which the peroxidation of phospholipids causes plasma membrane rupture (Dixon et al., 2012). This can be combated by adaptations in plasma membrane repair mechanisms dependent on the endosomal sorting complexes required for transport III (ESCRT-III) leading to resistance to ferroptosis as shown in pancreatic and liver cancer cell lines and *in vivo* (Dai et al., 2020). The ESCRT complexes mediate multiple membrane trafficking and remodelling processes, many of which might also contribute to tumorigenesis and tumour progression (see Box 2). Although many cancer cells adapt to evade apoptosis by upregulating anti-apoptotic proteins, ferroptosis is often lethal for cancer cells; indeed, drugs that increase lysosomal release of free iron to induce ferroptosis have shown promising results in pancreatic cancer models *in vitro* and have the potential to provide a new way of killing cancer cells that are resistant to existing therapies (Antoszczak et al., 2022; Mai et al., 2017). Understanding the intricate balance of iron homeostasis in cancer cells, wherein trafficking of the transferrin receptor plays a pivotal role, reveals not only its crucial involvement in supporting biosynthesis and meeting energy needs but also its potential as a vulnerability of tumour cells. The complex interplay between iron-dependent processes, such as ferroptosis, and ESCRT-III-dependent plasma membrane repair mechanisms presents an intriguing avenue for developing innovative therapeutic strategies, particularly for cancer cells resistant to conventional therapies.

**Box 2. The ESCRT complex**

The endosomal sorting complexes required for transport (ESCRT) protein complex, comprising ESCRT-0, ESCRT-I, ESCRT-II, ESCRT-III, contributes to vesicular trafficking, cytokinesis, plasma membrane repair and autophagy, but less is known about its potential role in tumorigenesis (Bache et al., 2004; Bishop et al., 2002; Carlton and Martin-Serrano, 2007; Morita et al., 2007; Takahashi et al., 2019). The ESCRT complex mediates inward membrane budding and is crucial for formation of intraluminal vesicles (ILVs) at endosomes prior to recycling of vesicle contents. These processes affect the duration and amplitude of growth factor receptor signalling by influencing the time that receptors spend on the cell surface and in endosomes (Bendris and Schmid, 2017; Mellman and Yarden, 2013; Mosesson et al., 2008). Receptors continue to signal from endosomes until they are internalised in ILVs inside the multivesicular body (MVB) (Tanaka et al., 2008). The formation of MVBs is also mediated by the ESCRT complex and its accessory proteins Vps4A and Vps4B. The subsequent fusion of MVBs with lysosomes results in degradation of the receptors and dampening of oncogenic signalling. The ESCRT complex is also involved in membrane remodelling during cytokinesis, and loss of ESCRT-mediated cytokinetic abscission results in multinuclear cells and genomic instability that predisposes the cell to malignancy (Sadler et al., 2018). Finally, ESCRT proteins contribute to the cancer hallmark of resistance to cell death by mediating membrane remodelling at sites of plasma membrane damage, an important cell survival mechanism (Jimenez et al., 2014).

### Macropinocytosis fuels tumour growth and aggressiveness

Macropinocytosis is an important pathway for the internalisation of adhesion molecules, which regulate cancer cell invasion, and cholesterol, which plays a crucial role in hormone-dependent cancers and regulates pro-proliferative signalling through the mammalian target of rapamycin complex (mTORC) and Wnt pathways (Xu et al., 2020a), and contributes to reprogramming and increased invasion capability in breast cancer cells *in vitro* (Skorda et al., 2023). Although the primary pathway for cholesterol uptake is through low-density lipoprotein (LDL) receptor-mediated endocytosis, cancer cells can also internalise cholesterol via macropinocytosis using the scavenger receptor SR-B1 (also known as SCARB1) (Mooberry et al., 2016; Skorda et al., 2023). Normal regulation of cholesterol uptake involves transcriptional control of the LDL receptor to prevent excessive intracellular cholesterol concentrations, but SR-B1 bypasses these regulatory mechanisms. High expression levels of SR-B1 in prostate and breast cancer correlate with elevated intra-tumoral cholesteryl-esters, aggressive disease and poor outcome in patients (de Gonzalo-Calvo et al., 2015; Schörghofer et al., 2015; Yuan et al., 2016).

During invasion, mechanical forces are communicated to cell adhesions by changes in membrane curvature and cortical actin polymerisation (Caswell et al., 2009; Franceschi et al., 2015). This interplay between the cytoskeleton, membrane and signalling results in activation of Rac1; subsequent actin polymerisation drives membrane ruffling and formation of macropinosomes, which mediate cell adhesion and chemotaxis (Le et al., 2021; Veltman et al., 2016). By modulating macropinocytosis, cancer cells can regulate their invasiveness through trafficking of adhesion molecules such as integrins. An example is the protein CYFIP related Rac1 interactor A (CYRI-A), which regulates actin dynamics in the early stages of macropinocytic cup formation. Importantly, loss of CYRI-A promotes cancer cell migration and invasion *in vitro* as a result of decreased internalisation via macropinocytosis of the α5β1 integrin (Le et al., 2021). In summary, macropinocytosis supports tumorigenesis by regulating the uptake of cholesterol and adhesion molecules in order to sustain increased metabolism, invasion and migration in aggressive disease. These new insights into adaptations in cholesterol uptake pathways and the role of macropinocytosis in adhesion molecule internalisation provide valuable potential targets for therapeutic interventions aiming to modulate cancer invasiveness and progression.

### Trans-cellular endocytosis mobilises energy for senescent cancer cells

By arresting the cell cycle, cancer cells can enter senescence, rendering them resistant to chemotherapy-induced apoptosis, which can cause lethal disease relapse in patients (Ungerleider et al., 2018). These therapy-resistant ‘persister’ cells can drive relapse through secretion of chemokines and cytokines that promote proliferation, metastasis and survival *in vitro* and *in vivo* in a range of cancer types (Canino et al., 2012; Rodier et al., 2009; Toso et al., 2014). The senescent cell still retains a high metabolic burden, which is overcome through upregulation of genes encoding proteins that facilitate autophagy and phagocytosis (Farrow et al., 2014; Itkonen et al., 2017; Mukhopadhyay et al., 2023; Tonnessen-Murray et al., 2019; Xu et al., 2020b). However, autophagy only provides a limited source of nutrients (Xu et al., 2020b). Recent research shows that senescent cancer cells use their phagocytosis machinery and actin cytoskeleton to engulf neighbouring tumour cells and take up amino acids and lipids that can fuel metabolism and provide increased cell viability (Tonnessen-Murray et al., 2019). This process, termed trans-cellular endocytosis, is regulated by elevated levels of the phosphoinositides phosphatidylinositol 4-phosphate [PI(4)P] and phosphatidylinositol 4,5-bisphosphate [PI(4,5)P2] at the plasma membrane and the endocytic coat protein clathrin (see Box 1) (Frey et al., 2022).

In summary, metabolic reprogramming is now an established hallmark of cancer, and has been demonstrated to be significantly sustained by adaptations in membrane trafficking at a number of levels including nutrient uptake, nutrient storage and nutrient degradation and turnover.

## Modulation of ER-to-Golgi trafficking drives cancer progression

Increased expression of components of the COPII coatomer complex, which drives ER-to-Golgi anterograde vesicle trafficking, has been associated with a range of cancers (Table 1). The capacity of the COPII trafficking pathway is important for alleviating ER stress and proteotoxicity arising from drivers of tumorigenesis that enhance rates of protein synthesis, such as loss of PTEN and activation of Myc (Biffo et al., 2018; Sellars et al., 2019; Zhang et al., 2017). Interestingly, in some cancer contexts, decreased expression or activity of COPII components can also promote tumour growth and invasion by altering trafficking of cell surface receptors and adhesion molecules. Here, we will explore how COPII vesicle trafficking is adapted in different cancer types to sustain tumorigenic processes.

### Crosstalk between the unfolded protein response and COPII vesicle trafficking confers resistance to ER stress

Upregulation of COPII coatomer proteins in cancer is sustained by the activation of ER stress-responsive transcription factors: for example, transcriptional regulation of the ER-to-Golgi vesicle pathway by overexpression of the transcription factors CREB3L2 and CREB3 has been reported both in prostate and breast cancer and correlates with disease progression (Howley et al., 2018; Hu et al., 2021). CREB3L2 directly regulates the transcription of COPII vesicle coat proteins SEC13, SEC23A, SEC24D and SEC31A, and studies have shown enhanced trafficking from the ER-to-Golgi associated with upregulated CREB3L2 in prostate cancer cells using the retention using selective hooks (RUSH) assay (Hu et al., 2021; Khetchoumian et al., 2019; Tomoishi et al., 2017).

Activation of inositol-requiring enzyme 1 α (IRE1α) and its downstream transcription factor X-box binding protein 1 (XBP1) also accelerate ER-to-Golgi trafficking in the liver (Liu et al., 2019a). The IRE1α-XBP1 signalling pathway is a major branch of the unfolded protein response (UPR) to ER stress. Activation of this pathway helps limit proteotoxic stress by increasing the expression of chaperones such as heat shock proteins (e.g. HSP90 family proteins) and, in the case of XBP1, protein disulphide isomerases (Hendershot et al., 2023). In prostate cancer, the androgen receptor (AR) was shown to transcriptionally activate IRE1α and XBP1 to sustain tumorigenesis through increased rates of anabolic metabolism and lipid turnover (Sheng et al., 2015). In gastric cancer, SEC23A expression is increased as a result of activation of the pro-inflammatory transcription factor STAT3 by phosphorylation at tyrosine-705, which is induced via the protein kinase RNA-like ER kinase (PERK)-mediated branch of the UPR (Cheng et al., 2023). In gastric and prostate cancer cells, the suppression of COPII coatomer components or their regulating UPR-associated transcription factors through genetic manipulation or therapeutic targeting (in the case of the IRE1α-XBP1 axis) results in apoptosis (Cheng et al., 2023; Sheng et al., 2015). This is due to the induction of autophagy downstream of PERK-mediated UPR signalling through the transcription factor ATF4, which sustains the expression of pro-apoptotic factors such as DNA damage-inducible transcript 3 (DDIT3) and TNF-related apoptosis-inducing ligand (TRAIL; also known as TNFSF10) receptor as well as autophagy genes such as ATG5 (Rashid et al., 2015).

Interestingly, COPII coatomer subunits can also alleviate ER stress through COPII pathway-independent mechanisms. For example, SEC23B has been reported to relocate to nucleoli under conditions of ER stress and contribute to combating ER stress by disrupting ribosomal RNA (rRNA) processing, thus restricting ribosome biogenesis and protein synthesis (Yehia et al., 2021). SEC23B can also act non-canonically in the nucleus to sustain increased rates of rRNA transcription by interacting with upstream binding transcription factor (UBF), a key protein of the transcription pre-initiation complex, to mediate RNA polymerase I (Pol I) recruitment to ribosomal DNA (rDNA) promoter regions (Yehia et al., 2015). These non-canonical functions are most prominent when SEC23B is mutated (e.g. V594G) as found in Cowden syndrome, which is an autosomal dominant disorder characterised by multiple hamartomas and an increased lifetime risk of cancer, with epithelial thyroid carcinoma being a major clinical component (Yehia et al., 2015).

Thus, mutations and altered expression of COPII coatomer subunits in cancer cells are associated with an increased capacity to withstand ER stress under conditions of enhanced protein synthesis and anabolic metabolism. Many cancers associated with alterations in COPII pathway activity arise from cell types that possess specialised secretory functions in glandular organs; for example, luminal epithelial cells in the prostate gland, and oesophageal and breast cancers (Du et al., 2023; Howley et al., 2018; Hu et al., 2021; Liu et al., 2019b). These cancers therefore retain specialised membrane trafficking functions associated with their cell types of origin in order to support cancer cell survival.

### COPII vesicle trafficking mediates cell surface expression of RTKs and adhesion molecules

Rather than by increasing the capacity to tolerate ER stress, COPII mutations in cancers that do not arise in cells with specialised secretory functions, such as colorectal cancer, support tumorigenesis by impacting on the cell surface activity and expression of growth factor receptors and epithelial markers. For example, in colorectal cancer, mutation or decreased expression of the COPII coat protein SEC23B impairs intracellular trafficking, resulting in reduced protein transport of epithelial cell adhesion molecule (EPCAM) to the plasma membrane and increased metastatic progression (Yang et al., 2020). Another example involves the epidermal growth factor receptor (EGFR), which is a tyrosine kinase receptor located at the cell surface that is commonly activated in cancers and promotes proliferation and tumorigenesis. Aberrant EGFR trafficking and glycosylation is linked to resistance to therapeutic tyrosine kinase inhibitors, which are critical treatment options for breast and non-small lung cancer (Sambrooks et al., 2018; Scharaw et al., 2016). EGFR is highly N-glycosylated in the ER and Golgi, and its transport to the plasma membrane is sensitive to aberrant glycosylation and expression of the COPII inner coat complex proteins SEC23B, SEC24B and SEC24D (Dragic et al., 2022; Scharaw et al., 2016).

These examples collectively offer an enhanced understanding of the advantages gained by tumour cells through the augmentation or alteration of COPII-mediated protein trafficking and secretion. This not only enables efficient ER-to-Golgi trafficking of proteins involved in promoting invasion and proliferation, but also aids tumour cells in coping with ER stress arising from an increased demand for protein synthesis. The elevated capacity for secretion and trafficking observed in various cancers thus establishes an efficient and robust pathway to facilitate pro-proliferative signalling, migration and resistance to apoptosis.

## Addiction to Golgi-to-ER trafficking promotes cell survival and tumorigenesis

**Fig. 2.**
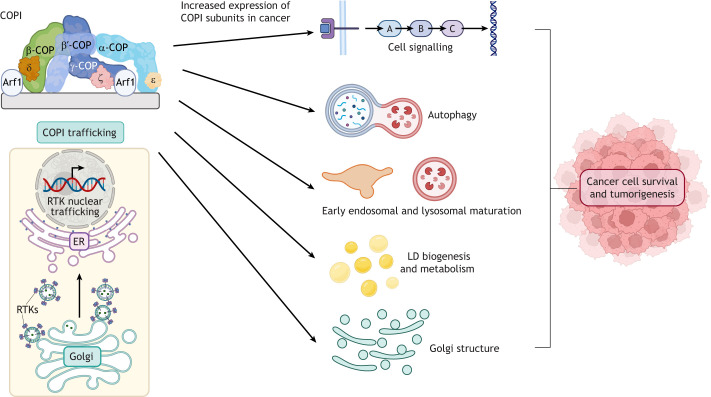
**Changes in COPI coatomer expression promote cancer cell survival and tumorigenesis.** The COPI complex consists of seven coatomer units: α, β and β′, γ, δ, ε and ζ. The γ−ζ−β−δ subunits form one arch-like structure whereas the α and β′ subunits dimerise to form a second arch-like structure. Assembly of COPI is regulated by the membrane-associated small GTPase Arf1, which regulates cargo sorting, vesicle scission, and vesicle uncoating during cargo delivery (Taylor et al., 2023). COPI-coated vesicles traffic from the Golgi to the ER and are crucial for regulation of cell signalling pathways, autophagy, the endolysosomal pathway, lipid droplet (LD) biogenesis, metabolism and maintaining the structure of the Golgi. COPI-mediated trafficking is also crucial for retrograde transport of RTKs to the nucleus, where they regulate expression of target genes. In cancer, COPI subunits frequently display increased expression, which enhances both the trafficking capacity of this pathway and the retrograde nuclear transport of receptor RTKs that promote oncogenic signalling (Taylor et al., 2023), thus aiding cancer cell survival and tumorigenesis through enhanced pro-proliferative signalling, improved nutrient availability during growth, and sustained trafficking and modification (e.g. glycosylation) of proteins in the Golgi.

A decade ago, it was shown that COPI coatomer expression and retrograde Golgi-to-ER trafficking sustain lysosomal activity and metabolic function in *KRAS* and *LKB1* (also known as *STK11*) co-mutant lung cancers (Fig. 2) (Kim et al., 2013). There is now evidence that COPI vesicle trafficking promotes cell survival and tumorigenesis in breast, lung, prostate and thyroid cancers, as well as in other cancer types (Bhandari et al., 2019; Claerhout et al., 2012; Feng et al., 2021; Kim et al., 2013; Marco et al., 2020). Differential gene expression analysis of cancer tissue and cell line data sets has revealed that genes encoding α- and β-COPI coatomer subunits are highly expressed in seven cancer types – breast, ovarian, prostate, colorectal, mesothelioma, bladder and lung cell cancer (Claerhout et al., 2012; Kim et al., 2013; Mi et al., 2016; Pu et al., 2018; Sudo et al., 2010) (Table 1). Moreover, high expression levels of the COPI β-subunit gene *COPB2* correlate with poor survival for breast (Pawitan et al., 2005), prostate (Mi et al., 2018) and ovarian cancer (Berchuck et al., 2005). Additionally, proteomic studies have shown that COPI coat proteins are more highly expressed in prostate cancer tissue samples than in normal prostate tissue (Latonen et al., 2018). Next, we will discuss the specific mechanisms by which tumour cells adapt COPI-mediated trafficking to promote cell survival and proliferation.

### COPI trafficking regulates autophagy

Recent findings support a role for COPI-coated vesicles in the regulation of autophagy (Claerhout et al., 2012; Gasparian et al., 2022; Shtutman and Roninson, 2011; Stewart et al., 2021). Autophagy has been observed in tumours to help sustain metabolic activity and cell survival in adverse, nutrient-poor environments; for a comprehensive review of the role of autophagy at different stages of cancer development, see Debnath et al. (2023). In prostate cancer cells, reduced expression of the α, β, δ or ζ COPI subunits leads to increased apoptosis and accumulation of autophagic markers p62 (also known as SQSTM1) and LC3-II (the lipidated form of MAP1LC3 proteins) (Feng et al., 2021; Gasparian et al., 2022; Stewart et al., 2021). In line with these findings, experimental evidence suggests that COPI-mediated vesicle trafficking promotes cancer cell survival and tumorigenesis by maintaining the morphology of the Golgi and sustaining a range of cellular processes, such as autophagy, lipophagy and downstream signalling pathways (Claerhout et al., 2012; Gasparian et al., 2022; Stewart et al., 2021). Depletion of the COPI complex in prostate cancer cell lines results in an accumulation of lipid droplets that colocalise with autophagy markers, indicating that loss of COPI inhibits lipophagy and metabolism of lipids (Gasparian et al., 2022). Understanding how COPI vesicle trafficking contributes to lysosomal degradation, autophagy and lipid metabolism has thus provided new insights to how overexpression of COPI proteins in tumour cells, on one hand, provides a survival advantage, but on the other hand offers the potential for therapeutic targeting of COPI components.

### COPI vesicle trafficking regulates lipid homeostasis

Altered lipid metabolism promotes proliferation, metastasis and anti-apoptotic mechanisms, and is a hallmark of several cancer types (Butler et al., 2016; Ward et al., 2021; Zhang et al., 2020a). As discussed above, cancer cells adapt to their increased need for energy through mechanisms including increased uptake of lipids. Such metabolic reprogramming is crucial for tumorigenesis and has been associated with increased lipogenesis and storage of fatty acids and cholesterol in lipid droplets, which mitigate against lipotoxicity and protect cancer cells from cell death (Petan, 2020; Sainero-Alcolado et al., 2024). Notably, COPI-mediated retrograde trafficking regulates lipid storage and cholesterol homeostasis through transcriptional regulation and trafficking of the lipase ATGL (also known as PNPLA2), which catalyses the initial step in triglyceride lipolysis, to the surface of lipid droplets (Beller et al., 2008; Guo et al., 2008; Soni et al., 2009; Takashima et al., 2015), as well as through trafficking of sterol regulatory element-binding proteins (SREBPs). SREBPs are sterol-sensing proteins that are trafficked from the ER to the Golgi when cholesterol concentrations are low (Radhakrishnan et al., 2007). In the Golgi, SREBPs are proteolytically processed and trafficked to the nucleus where they act as transcription factors and promote expression of genes that regulate fatty acid and cholesterol synthesis (Ding et al., 2018; Gu et al., 2022; Kuzu et al., 2016). Importantly, impaired COPI trafficking promotes activation and nuclear translocation of SREBPs resulting in upregulation of genes that promote lipid synthesis (Takashima et al., 2015). Thus, the adaptive strategies of cancer cells involving increased COPI vesicle proteins (Table 1) requires further investigation as the crosstalk with lipid homeostasis might play a pivotal role in metabolic reprogramming, promoting tumorigenesis and facilitating lipogenesis. Notably, the regulation of lipid storage and cholesterol homeostasis by COPI-mediated retrograde trafficking of factors such as ATGL and SREBPs underscores that retrograde trafficking adaptations fuel aberrant lipid metabolism, which in turn drives proliferation, metastasis, and anti-apoptotic mechanisms – a hallmark across various cancer types.

### COPI-mediated trafficking of receptor tyrosine kinases to the nucleus facilitates oncogenic signalling

COPI vesicle trafficking is also an important regulator of the retrograde trafficking of RTKs, including EGFR, ErbB (also known as HER2 and ERBB2) and Met receptors to the nucleus in breast and prostate tumours (Chen et al., 2019; Li et al., 2011; Wang et al., 2004, 2010; Xie et al., 2014; Zhang et al., 2019a). RTKs that localise to the nucleus have non-canonical substrates that promote oncogenesis by enhancing cell proliferation, metastasis, DNA repair, cancer progression and therapy resistance (for a complete review, see Chen et al., 2020). For example, the cancer-associated EGFR variant EGFRvIII is also a substrate of wild-type EGFR, and its phosphorylation allows EGFRvIII to associate with and subsequently activate STAT3 in the nucleus, resulting in oncogenic signalling (Fan et al., 2013). Retrograde trafficking of RTKs therefore has emerged as an interesting therapeutic target in triple-negative breast cancer. An inhibitor of retrograde trafficking, Retro-2 (Stechmann et al., 2010), was shown to inhibit cancer cell growth and has shown promising results *in vitro* and in *in vivo* mouse models (Madera et al., 2022).

In summary, COPI-mediated retrograde trafficking regulates a range of cellular activities that promote tumorigenesis by sustaining metabolism, energy homeostasis and pro-proliferative signalling. Although there is strong evidence for overexpression of COPI subunits in multiple kinds of cancer, the different mechanistic advantages this provides for cancer progression are still being uncovered. Current evidence suggests that COPI regulates autophagy, lysosomal activity, lipid homeostasis and retrograde transport of EGFR in cancer cells, and future research should focus on generating additional insights into the specific impacts of overexpression of COPI in a range of cancer subtypes.

## Adaptations in membrane trafficking impact responses to cancer therapies

Evasion of cell death through intrinsic and/or extrinsic apoptosis is another hallmark of cancer cells (Hanahan, 2022). Chemotherapy and radiotherapy are common treatment options for many cancer patients; in addition, in recent years cancer immunotherapy has become an important treatment avenue (Pasello et al., 2020; Sperandio et al., 2021). However, all cancer treatments are associated with mechanisms of resistance and recurrence, and it is therefore essential to understand the mechanisms underlying adaptations that allow cancer cells to resist cell death (Gutwillig et al., 2022; Jayashankar and Edinger, 2020; Tonnessen-Murray et al., 2019). Here, we will explore resistance mechanisms associated with two major types of chemotherapy that target dividing cells by impeding nucleotide synthesis or inducing DNA crosslinking. First, we will look at 5-fluorouracil (5-FU), which is employed in treating colorectal, breast, and pancreatic cancers by impeding DNA synthesis, reducing production of thymidine (an essential DNA component) and disrupting DNA repair processes, ultimately leading to programmed cell death. Second, we examine platinum-based chemotherapy (e.g. cisplatin and carboplatin), which is utilised in treating ovarian, bladder, lung and oesophageal cancers, and has a mechanism of action involves crosslinking of purine bases on DNA, which interferes with DNA replication and causes DNA damage, consequently triggering apoptosis. Finally, we will discuss resistance mechanisms associated with emerging cancer immunotherapy treatments, which promote activity of T cells against cancer cells.

### Resistance to chemotherapy-induced apoptosis via endocytic adatpations

**Fig. 3.**
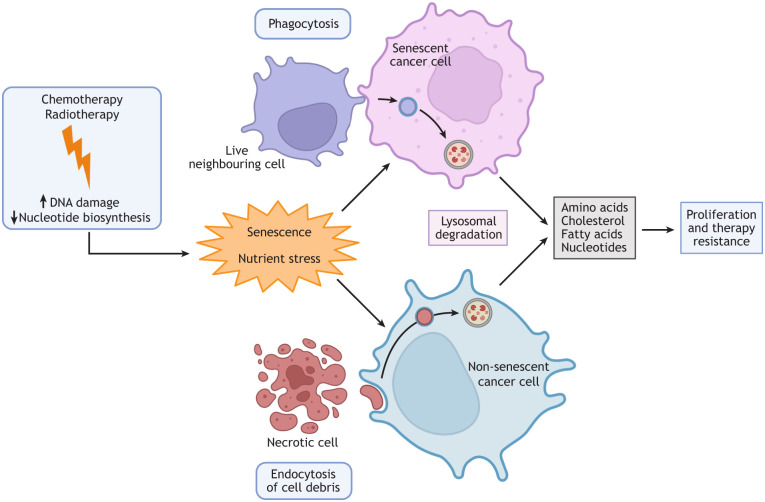
**Schematic illustration of the role of endocytosis in cancer cell survival and therapy resistance.** Major traditional chemotherapy and radiotherapy approaches induce DNA damage and inhibit nucleotide biosynthesis, which can trigger resistance mechanisms in cancer cells to promote survival. One such response is for cells to become senescent by arresting the cell cycle. Through adaptations to endocytic mechanisms, senescent cancer cells can bypass the effects of nutrient stress and lack of available nucleotides induced by chemotherapy, allowing them to maintain energy homeostasis and reduce the need for nucleotide biosynthesis. To survive, senescent cells might internalise parts of, or even entire living neighbouring cells, to sustain metabolism (top). Alternatively, cell debris from necrotic cells can be endocytosed by non-senescent cancer cells to retrieve nutrients and nucleotides (bottom).

As discussed above, cancer cells can resist chemotherapy-induced apoptosis by entering senescence. Senescent cancer cells can form a cell population that can reinitiate proliferation and form more aggressive and tumorigenic clones (Saleh et al., 2020; Tonnessen-Murray et al., 2019). By endocytosing and degrading membranes, protein and DNA from a live neighbouring cell, senescent cells can generate a pool of lipids, amino acids and nucleotides and bypass the intrinsic synthesis pathways that might be subject to inhibition by chemotherapies (Fig. 3) (Tonnessen-Murray et al., 2019). This mechanism is important in treatment resistance, as many chemotherapies induce cell death through nutrient stress by impairing nucleotide biosynthesis (e.g. 5-FU) or by causing DNA damage and creating a subsequent increased need for nucleotide synthesis (e.g. platinum-based therapies) (Brown et al., 2017; Heiden and DeBerardinis, 2017). By-products from dead cells can also be endocytosed and degraded by prostate and breast cancer cells (Jayashankar and Edinger, 2020; Kim et al., 2018; Tonnessen-Murray et al., 2019), which contributes to increased survival due to internalisation of fatty acids, nucleotides and amino acids from cell corpses. Thus, by scavenging nutrients, lipids and nucleotides, cancer cells become less dependent on and ultimately resistant to inhibition of the biosynthetic pathways that are targeted by 5-FU and platinum-based chemotherapies (Davidson and Heiden, 2017; Davidson et al., 2017; Jayashankar and Edinger, 2020; Palm et al., 2015). In other words, by using endocytosis and macropinocytosis, cancer cells can bypass the biosynthetic pathways that are targeted by classical chemotherapeutics because the end-products are instead taken up from an external supply (Jayashankar and Edinger, 2020; Labi and Erlacher, 2015).

### Cholesterol-mediated resistance to chemotherapy-induced apoptosis

Mechanisms of resistance against chemotherapy-induced apoptosis can also occur at the intrinsic apoptotic pathway on the outer mitochondrial membrane (OMM). Induction of intrinsic apoptosis is dependent on permeabilisation of the OMM, resulting in the release of cytochrome *c* and activation of caspases. The membrane composition of the OMM is therefore an important regulatory mechanism of intrinsic apoptosis, because the release of cytochrome *c* depends on the ability of the OMM to accept insertion of membrane-associated proteins, such as caspase-8, the initiator of intrinsic apoptosis, and the pore-forming Bax–Bak complex (Lucken-Ardjomande et al., 2008). For example, trafficking of cholesterol to mitochondria increases the stiffness of the OMM and provides resistance to apoptosis induced by 5-FU or cisplatin in colorectal and oesophageal cancer cell lines (Akpinar et al., 2016; Holloway et al., 2024). Indeed, impaired LDL receptor-mediated endocytosis of cholesterol and fatty acids results in sensitisation of cancer cells to platinum-based chemotherapy (Chang et al., 2019; Guillaumond et al., 2015). The connection between mitochondrial cholesterol and resistance to apoptosis and chemotherapy has been observed in multiple cancer types, and mechanisms involving both increased trafficking of cholesterol both to and from the mitochondria have been observed (Garcia-Ruiz et al., 2021; McCabe et al., 2022; Nguyen et al., 2022; Smith and Land, 2012). However, further work is required to elucidate the pathways *in vivo.* Nevertheless, these recent advancements in comprehending the involvement of membrane trafficking mechanisms in chemotherapy-induced apoptosis open up possibilities for developing therapies aimed at patients who have developed resistance, who remain a particularly challenging group to treat.

### ESCRT-mediated resistance to immunotherapy-induced plasma membrane damage

Cancer immunotherapy has progressed significantly in recent years and has proven successful in the treatment of liver, lung and skin cancer (Fletcher and Johnson, 2024; Iams et al., 2020; Pasello et al., 2020; Sperandio et al., 2021). Immunotherapy uses inhibitory antibodies to target immune checkpoint inhibitors, such as PD-L1 (also known as CD274), PD-1 (PDCD1) and CTLA-4, that normally switch off the immune response of cytotoxic T cells against cancer cells. In this response, perforin released from T cells forms pores in the plasma membrane of the cancer cell, which imports cytotoxic proteins (granzymes) that induce apoptosis. However, based on *in vitro* and *in vivo* experiments, it has been proposed that multiple attacks from T cells are required in order to achieve a lethal concentration of granzyme in tumour cells, which can take from one up to several hours (Weigelin et al., 2021). Following pore formation, an influx of Ca^2+^ triggers a rapid membrane repair response (Ritter et al., 2022). This response was found to involve recruitment of the ESCRT proteins Tsg101 and Chmp4b within 30 to 60 s after pore formation (Ritter et al., 2022). Furthermore, inhibition of the ESCRT complex sensitised cells to perforin- and granzyme-mediated cell death (Ritter et al., 2022). These exciting findings demonstrate a role for ESCRT proteins in resisting cell death associated with plasma membrane disruption in cancers in which these proteins are overexpressed (Neggers et al., 2020), and provide a rationale for their use as biomarkers in personalised therapy for treatment-resistant cancers (Box 2).

## Conclusion and future perspectives

Our knowledge of how cancer cells adapt membrane trafficking to promote tumorigenesis, growth and resistance to therapy is growing and will undoubtedly lead to discoveries that can help in treatment decisions, delivery of targeted therapies and consequently improved outcomes for patients. Membrane trafficking is a dynamic process, as are all processes that drive tumorigenesis. The role of membrane trafficking in cancer therapy resistance is only beginning to unfold and going forward, it will be important to evaluate the membrane trafficking capacity of cancers against snapshot or steady-state measurements of metabolites, receptor abundance or lipid composition in order to further delineate mechanisms of resistance related to membrane trafficking. Two areas of innovation will accelerate this: first, extended maintenance of patient tissue samples *ex vivo* as a viable pre-clinical/laboratory model; and second, improved capacity to perform rapid, high-resolution and multi-parametric imaging on thin and full-thickness tissue sections to visualise vesicle trafficking, phagocytosis and the subcellular location of protein complexes (Liu et al., 2023; Tagliatti and Cortese, 2022). Progress in these areas will enable us to better define the functional relationships between, for example, emergent prognostic lipid signatures in colorectal (Shimolina et al., 2021; Sun et al., 2022), prostate (Butler et al., 2021; Lin et al., 2017) and breast cancer (Chen et al., 2016; Purwaha et al., 2018), and the various membrane trafficking processes that might sustain those signatures in each cancer type. Such advances will enable us to more effectively stratify patients for treatment and provide functional contexts for lipid, protein and transcript signatures.

Understanding the roles of membrane trafficking in the efficacy of emerging cancer therapies also has the potential to guide and refine development of new treatments. Development of antibody–drug conjugates (ADCs), which are designed to deliver chemotherapeutic drugs directly to a tumour by targeting receptors specifically expressed on cancer cells, is a very active field in cancer therapy research. Unlike traditional chemotherapies, the premise for the efficacy of ADCs hinges on cell surface accessibility of the receptor target for on-target delivery of a radiotherapy or chemotherapy ‘warhead’, rather than on the capacity of the cell to take up the ADC. An advantage of ADCs is the attractive mitigation of off-target toxicity to healthy cells, but the challenges to implementing this treatment strategy include resistance mechanisms in which cell surface expression of the receptor target is downregulated or intracellular trafficking of the free drug is impaired (Beck et al., 2017; Khongorzul et al., 2020; Thomas et al., 2016). The adaptations in membrane trafficking described above provide some future directions for development of ADCs and targeted therapy. Developing tissue-based assays of the binding capacity of an ADC to the target cancer as well as the endocytic and endo-lysosomal trafficking capacity of the cancer cells will improve both patient selection and predictions of their response to treatment. The endo-lysosomal system and degradation of the ADC in the lysosome could be particularly important for releasing the free drug into the cytosol, where it can bind to a cytosolic target or traffic to a nuclear targets. These factors are equally significant to consider for the use of nanoparticles as drug delivery vectors to enhance chemotherapy-induced cell death (Chu et al., 2022; Zhu et al., 2022).

We are now entering a new era in which functional cell biology will be able to enhance treatment decision-making and patient prognostication, and a better biomedical understanding of membrane trafficking as a dynamic process is well-positioned to significantly benefit cancer patients. Although altered membrane trafficking is not currently regarded as a cancer hallmark, evidence increasingly suggests that it deserves to be accorded that status as a vital enabling adaptation driving cancer cell metabolism and treatment resistance.
